# ESTES recommendation on thoracolumbar spine fractures: January 2023

**DOI:** 10.1007/s00068-023-02247-3

**Published:** 2023-04-13

**Authors:** Klaus Wendt, Christoph Nau, Marko Jug, Hans Christoph Pape, Richard Kdolsky, Sam Thomas, Frank Bloemers, Radko Komadina

**Affiliations:** 1 University Medical Center Groningen, University of Groningen, Groningen, The Netherlands; 2 University Hospital Frankfurt, Goethe University, Frankfurt, Germany; 3 University Medical Centre Ljubljana, University of Ljubljana, Ljubljana, Slovenia; 4 University Hospital of Zürich, University of Zürich, Zürich, Switzerland; 5 University Clinic for Trauma Surgery, Medical University of Vienna, Vienna, Austria; 6 Jan Yperman Hospital, Ypres, Belgium; 7 Amsterdam University Medical Centre, Vrije Universiteit Amsterdam, Amsterdam, The Netherlands; 8 Medical Faculty, University of Ljubljana, Ljubljana, Slovenia

## Chapter 1: Introduction

Spinal trauma is less common than other musculoskeletal injuries, yet leads to more disability and costs. In recent decades reliable classification and injury assessment systems have been published and surgical techniques have greatly improved. There are many new insights into the principles and timing of the treatment of thoracolumbar injuries, but many unsolved problems remain:

1. Role and timing of medical and surgical interventions for patients with associated neurological injury.
2. Timing of surgical intervention in patients with multiple injuries.
3. Wide variation in practice between operative versus non-operative management, without clear reasons.
4. The role of different surgical approaches and techniques in certain injury types is not clarified yet.
5. Methods of non-operative management.
6. No consensus is found yet for the care of elderly patients with concurrent complex disorders [1].

After initial assessment and management, a decision about the definitive treatment has to be made. There are two possibilities: conservative or operative treatment. The decision depends on the severity of the fracture (classification), spinal cord injury and possible comorbidity, and must be made together with the patient (shared decision-making). There are many different ways to treat a patient, both conservative and operative. Because of the impact of thoracolumbar injuries and the many options for treating these injuries, the European Society of Trauma and Emergency Surgery established a working group on this topic. The members are:

Frank Bloemers: The Netherlands.

Marko Jug: Slovenia.

Richard Kdolsky: Austria.

Radko Komadina: Slovenia.

Christoph Nau: Germany.

Hans Christoph Pape: Switzerland.

Sam Thomas: Belgium.

Klaus Wendt: The Netherlands.

Every working group member took care of one or two chapters. The content of the chapters is based on recent literature. In June 2022 a consensus meeting took place in Frankfurt and all chapters were discussed, resulting in consensus on all topics. After the meeting this draft was edited by Klaus Wendt and sent to all members for final comments.

## Chapter 2: Prehospital and emergency room management

The initial management of patients with thoracolumbar spine (TLS) injuries starts at the scene in accordance with emergency care treatment protocols (e.g. Advanced Trauma Life Support and/or European Trauma Course) and established algorithms (e.g. ABCDE). In polytraumatized patients or patients with other dominant injuries and/or quantitative or qualitative consciousness impairment (intoxication, head injury) and/or spinal pain and/or neurological deficits (e.g. motoric and sensory deficits, priapism), full in-line spinal immobilization using a scoop stretcher and/or vacuum mattress or spinal board including cervical and head immobilization is required at the injury site and maintained until a spinal injury is ruled out in the emergency room [2]. To prevent skin problems, immobilization devices should not be used indiscriminately and/or for prolonged periods [3].

Patients with a TLS injury, especially with neurological involvement, should be transported directly to a hospital where definitive spinal care can be achieved, but in case of concomitant life-threatening injuries transport to the nearest hospital capable of handling life-threatening injuries is indicated. During transport patient data (age and sex, time and mechanism of injury, vital signs, identified injuries, neurological impairment and therapeutic measures) and estimated time of arrival should be reported systematically [4].

After admission to the emergency room the management depends primarily on injury severity. In case of multiple injuries and/or neurological injury the patient should be treated by a resuscitation team according to protocols and invasive monitoring should be started immediately. After initial stabilization a secondary survey should include a more detailed clinical examination and neurological evaluation (e.g. ASIA Impairment Scale). A whole-body CT Angiography scan is suggested to identify or rule out potential life-threatening and spinal injuries. Life-threatening injuries must be treated first and treatment of spinal injuries must follow patient stabilization, but the time delay from injury to treatment should not be postponed for non-medical reasons, especially in case of neurological involvement. The decision to use corticosteroids in patients with spinal cord injury is at the discretion of the attending physician. However, one should be aware of the possibility of respiratory and intestinal tract complications, therefore the routine use of high-dose corticosteroids is not recommended [5].

In cases of an isolated spinal injury without neurological involvement urgent surgical treatment is usually not needed. Special attention in the emergency room is also required for patients older than 65 and patients with known osteoporosis or spinal disease (e.g. ankylosing spondylitis, diffuse idiopathic skeletal hyperostosis, tumour, infection), with complaints of back pain, and/or with high-energy injuries (e.g. fall from a height, high-speed motor vehicle or motorcycle accident) [2].

## Chapter 3: Diagnostics

Assessment of the patient should follow the primary and secondary survey in conformity with the ATLS principles. The physical examination should be followed by imaging.

### Conventional radiography

**Fig. 1:**
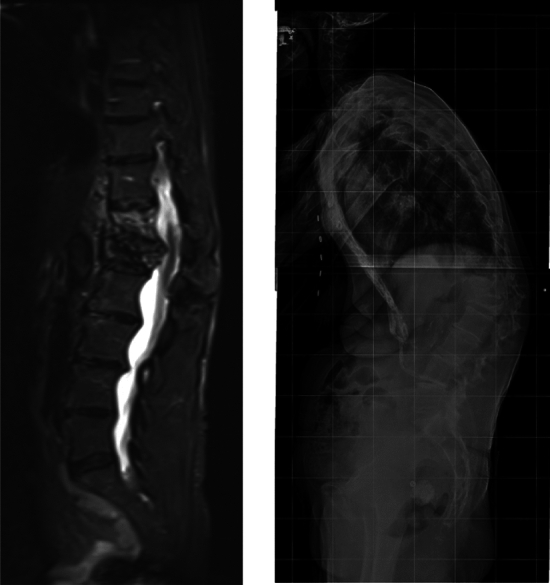
**A** MRI showing T12 and L1 burst fractures in a recumbent position. **B** standing full-spine radiograph showing massive kyphotic collapse of the fracture despite brace therapy

In most cases, X-rays in two plains are the first step in radiological diagnostics. However, up to 30% of spinal fractures might go undetected or underestimated on radiographs, especially in the cervical and upper thoracic spine [6–10]. In patients who sustained injuries during low-energy trauma, conventional radiographs are still indicated, guided by the clinical findings. Radiographs in a standing position can be useful in surgical decision-making in the early phase during patient admission or early follow-up of conservative treatment, as this allows assessment of the true extent of kyphotic deformity of a vertebral fracture under loaded conditions. Mehta et al. demonstrated that the bisegmental kyphosis angles (sagittal Cobb angle) on standing radiographs were on average 7° higher than supine radiographs (see Fig. 1) [11]. Local post-traumatic kyphosis exceeding 20° is frequently associated with posterior ligamentous complex injury [12]. If a vertebral fracture was detected or could not be reliably ruled out, an additional computed tomography (CT) scan of the suspicious segment should be obtained [13–15].

### Computed tomography (CT)

Computed tomography (CT) has replaced X-rays in most trauma centres for the initial assessment of high-velocity trauma patients, as it allows for rapid evaluation of visceral as well as bony injury, expediting treatment [6–10]. Its sensitivity reaches almost 100%, and it provides details about vertebral fracture morphology (comminution), presence of bony (posterior wall) fragments in the spinal canal, and indirect signs of disruption of the posterior ligamental complex (PLC) [16–19]. All this allows for adequate classification of a particular fracture, and in turn appropriate treatment. Within the context of a high-velocity trauma, CT of the TL spine can be done in conjunction with that of head, neck, chest cavity and abdomen, and all this within a very limited time frame, allowing for expedited treatment of all detected injuries [6, 7, 10, 16].

### Magnetic resonance imaging (MRI)

MRI can provide additional information about discoligamentous injuries, especially in the PLC, occult fractures and spinal cord injuries [17, 20]. It allows differentiation between old and new osteoporotic fractures in the elderly, particularly in T2-weighted STIR images. In these cases bony oedema demonstrating a non-healed fracture can be detected [6, 16]. MRI can be useful in the diagnostic work-up of patients with ankylotic conditions after a relevant trauma, not only to obtain more information on the specific injury pattern of a given fracture but also to rule out epidural haematoma and occult fractures in patients with negative CT-scans and multi-level or multi-segment fractures, which occur in 6 to 8% of cases [16, 24, 25]. Patients with presumed spinal cord injury should undergo an MRI as soon as possible, as this test can reveal the location and severity of the lesion, and at the same time indicate the cause of spinal cord compression. This is especially useful in the management of patients with incomplete spinal cord injury, for whom surgical intervention may prevent further deterioration. Several types of traumatic spinal cord lesions can be found:

- Intramedullary haemorrhage.
- Spinal cord contusion/oedema.
- Extrinsic compression by a bone fragment.
- Traumatic disc herniation/complete transection of the cord.

Last, we need to point out that MRI can confirm spinal cord injury absent any abnormality on radiographs and even CT imaging, a condition known as spinal cord injury without obvious radiological abnormalities or SCIWORA [16].

## Chapter 4: Classifications

Fractures of the thoracolumbar spine have been classified by different authors. [26–33] Based on Denis’ three-column model [34], in 1994 Magerl et al. introduced the AO-Magerl classification differentiating between three types of fractures by trauma mechanism (A Compression, B Distraction, C Axial torsion/Rotation) with three subtypes each, based on the two-column model [35]. In 2005 Vaccaro et al. proposed the Thoracolumbar injury classification system (TLICS), which was similar to the AO-Magerl classification regarding injury morphology but also took neurological status and damage of the PLC into consideration [37]. Based on this, the Thoracolumbar Injury Severity Score was developed to guide surgical decision-making [38].

In 2013, a “new” AOSpine classification was published, merging key elements of the AO-Magerl classification and the TLICS. The fracture morphology distinguished between three main types with several subtypes (A compression injuries; A0 minor, nonstructual fractures; A1 wedge compression; A2 split; A3 incomplete burst; A4 complete burst. B distraction injuries; B1 transosseus tension band disruption/Chane fracture; B2 posterior tension band disruption; B3 hyperextension. C translational injuries/displacement or dislocation). It also proposed a five-step approach to evaluate the status (N0 neurology intact, N1 transient neurological deficit, N2 radicular symptoms, N3 incomplete spinal cord injury or any degree of cauda equine injury, N4 complete spinal cord injury, NX cannot be examined) while regarding continued spinal cord compression (+). The modifiers (M1-M2) address the presence of an injury of the posterior ligament complex (PLC) (M1) as well as patient-specific comorbidities (i.e. ankylosing spondylitis, polytrauma, osteoporosis, overlying burns) (M2) [39].

**Fig. 2:**
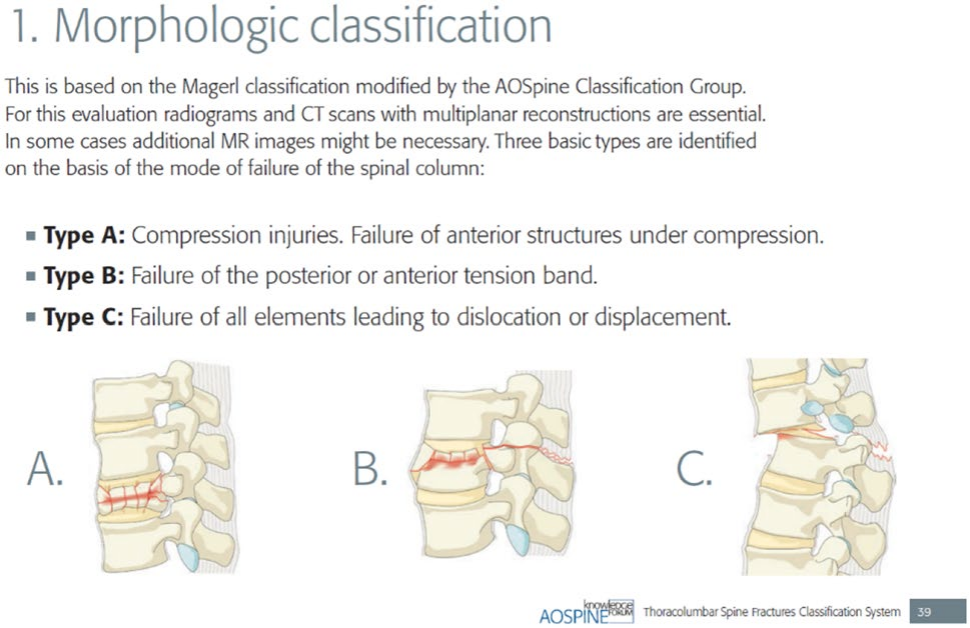
Morphologic classification

**Fig. 3:**
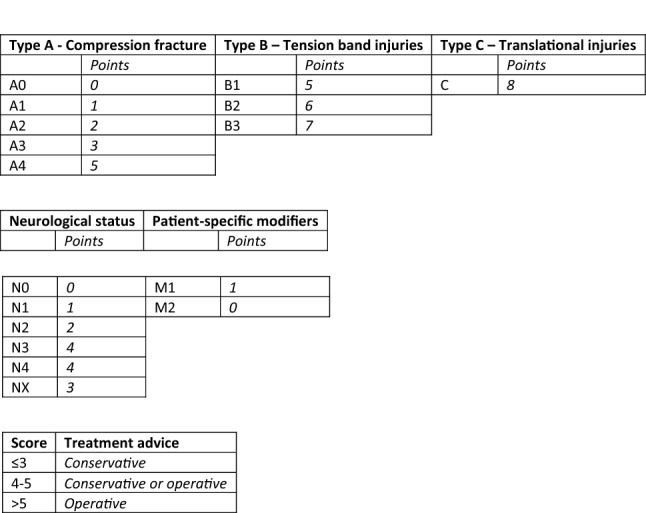
Thoracolumbar AOSpine injury score (TL AOSIS)(20)

Based on this classification system, the Thoracolumbar AOSpine injury score (TL AOSIS) was proposed to guide surgical decision-making, following the treatment advice by worldwide experts in spine surgery [40]. Reliability of the TL AOSIS is proven in numerous studies and has become an important tool for spinal surgeons everywhere [41–44]. Nowadays the AO classification is used almost exclusively, and the AOSpine Knowledge Forum Trauma is constantly optimizing it for use in clinical practice (Figs. 2, 3).

**Fig. 4:**
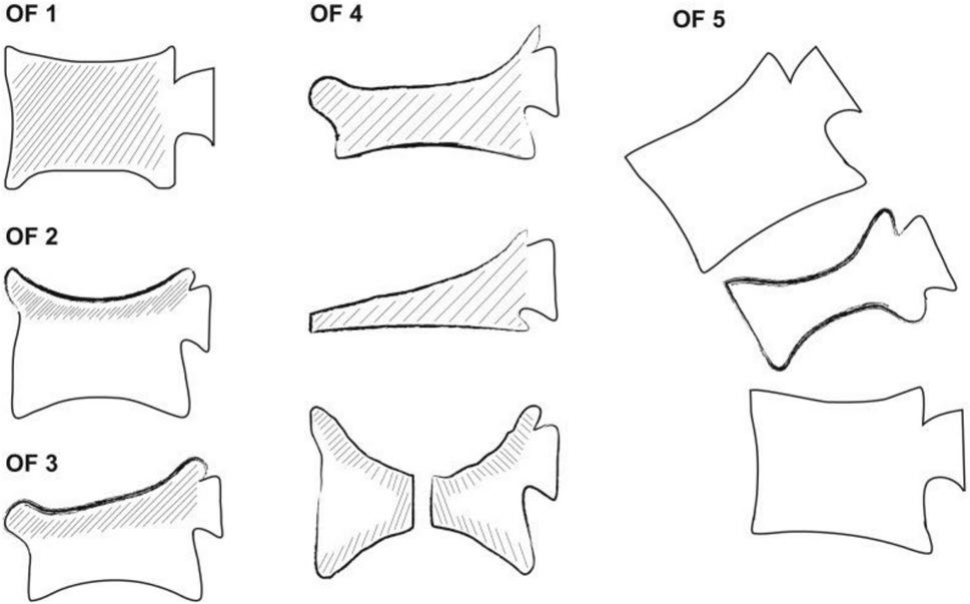
Osteoporotic fracture classification determined by the DGOU working group

Of note is that the incidence of osteoporotic fractures has increased in a sustained fashion. A working group has therefore developed a proposal for an osteoporotic fracture classification consisting of five groups that demonstrates the following features: OF 1, no vertebral deformation (vertebral oedema); OF 2, deformation with no or minor (< 1/5) involvement of the posterior wall; OF 3, deformation with distinct involvement (> 1/5) of the posterior wall; OF 4, loss of integrity of the vertebral frame, vertebral body collapse or pincer-type fracture; OF 5, injuries with distraction or rotation. The score is depicted in Fig. 4.

Schematic representation of the 5 OF subtypes (OF 1–5)

- OF 1: No vertebral deformation (vertebral body oedema in MRI-STIR only). This type is rare. The stable injury is clearly visible on MRI-STIR sequence only. X-rays and CT scan do not show vertebral deformation.
- OF 2: Deformation with no or only minor involvement of the posterior wall (< 1/5). This type of fracture affects one endplate only (impression fracture). There can be involvement of the posterior wall, but only minor. OF 2 are stable injuries.
- OF 3: Deformation with distinct involvement of the posterior wall (> 1/5). This type of fracture affects one endplate only, but shows distinct involvement of the anterior and posterior walls (incomplete burst fracture). The fracture can be unstable and may collapse further over time.
- OF 4: Loss of integrity of the vertebral frame structure, vertebral body collapse, or pincer-type fracture. This subgroup consists of three fracture types. In case of loss of integrity of the vertebral frame structure, both endplates and the posterior wall are involved (complete burst fracture). A vertebral body collapse is typically seen as a final consequence of a failed conservative treatment and can impose as a plain vertebral body. Pincer-type fractures involve both endplates and may lead to severe deformity of the vertebral body. OF 4 are unstable fractures and intravertebral vacuum clefts are often visible.
- OF 5: Injuries with distraction or rotation. This group is rare but shows substantial instability. The injury includes not only the anterior column but also the posterior bony and ligamentous complex. OF 5 injuries can be caused either by a trauma directly or by ongoing sintering and collapsing of an OF 4.

## Chapter 5: Non-operative treatment

### Introduction

The decision of whether a fracture of the thoracolumbar spine can be treated conservatively must be made based on a variety of criteria. Biological age, bone quality, activity level, individual requirements of the patient must be considered, plus the stability of the fracture as most important criterion [46].

A fracture is defined as stable if no neurological aggravation and no change in position are to be expected in the context of functional therapy. A fracture can be described as highly unstable if mobilization threatens a neurological aggravation.

Precise assessment of a fracture is crucial for optimal therapy [47]. According to the German Society of Orthopaedics and Trauma (DGOU), four morphological modifiers (MM) were introduced in addition to the AO Spine Classification [48, 49], derivable from conventional radiographs and CT images. These criteria clarify statements about the stability of the fracture and allow the possible treatment options to be derived [50, 51].

**Fig. 5:**
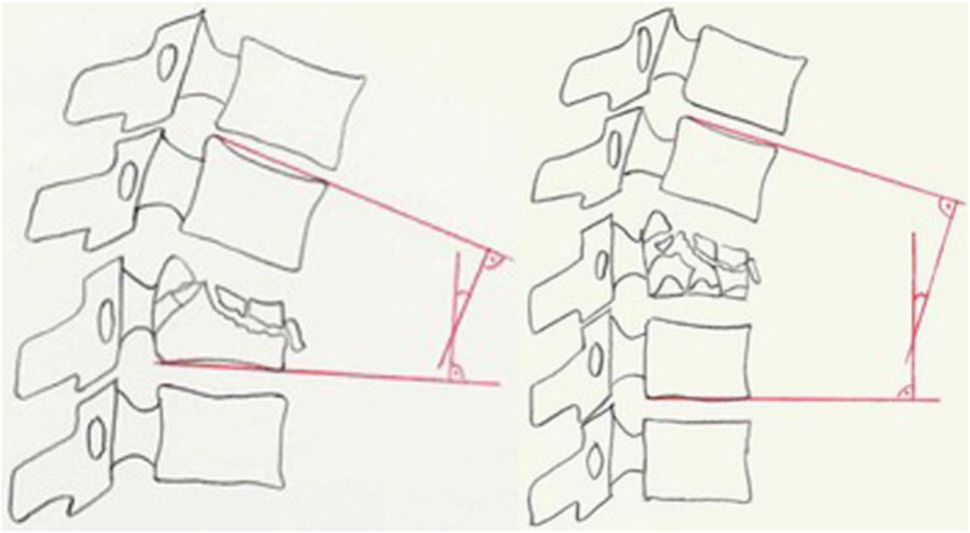
Morphological modifier 1 (MM 1): Disorder in the physiological alignment of the vertebral column: monosegmental and bisegmental endplate angle (EPA) (Spine Section DGOU [51])

**MM 1:** Deviation from the physiological profile of the spine: fractures can affect the physiological profile of the spine both in the sagittal plane (kyphosis/lordosis) and in the frontal plane (scoliosis). To describe this deviation, monosegmental and bisegmental endplate angles (EPA) are used in the sagittal plane [52]. The monosegmental and bisegmental scoliosis angles are used for description in the frontal plane. If the endplate of the injured vertebral body is involved, the bisegmental endplate angle (EPA) is used (Fig. 5).

The monosegmental scoliosis angle is used to describe changes in the frontal plane. This angle is formed by a straight line through the lower endplate of the injured vertebral body and through the upper endplate of the vertebral body above the injured vertebra. If the lower endplate of the injured vertebral body is involved, the bisegmental scoliosis angle should be used.

Decisive for the therapy is not only the measured angle but the deviation from the individual sagittal profile of the spine. For this reason, the difference between the physiological angle of curvature of the spine and the measured EPA is given as δ-EPA. It should be considered that the EPA can differ greatly between images taken in a standing and lying position. Whenever possible, images should be taken in the standing position. If a highly unstable fracture is suspected, initial standing radiographs are not recommended. The δ-EPA at the start of therapy allows conclusions to be drawn about the stability of the fracture and the therapy options. If a δ-EPA < 15–20° is present at the start of therapy in the standing position and the posterior column is intact, functional therapy under regular standing X-ray check-ups is the preferred treatment option.

**Fig. 6:**
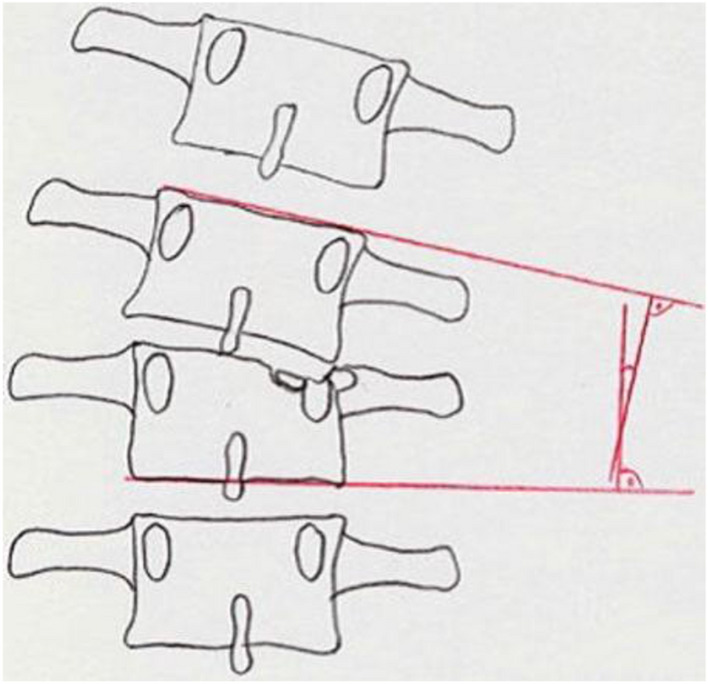
Morphological modifier 1 (MM 1): Disorder in the physiological alignment of the vertebral column: scoliosis angle (Spine Section DGOU [51])

If there is a scoliosis angle < 10° at the beginning of therapy while standing, functional therapy under regular standing X-ray check-ups is the preferred treatment option (Fig. 6).

**MM2**: Destruction of the vertebral body: The decision for surgical or conservative therapy and especially for ventral reconstruction is made largely based on the destruction of the vertebral body [53–55]. The destroyed volume of the vertebral body and the fracture dislocation are significant here. To assess the destroyed volume, the vertebra is divided into three equally large, horizontal thirds. A cranial, a medium and a caudal third are distinguished. It is described to what extent the volume of the vertebral body is affected by the fracture. Another criterion for an expectable increase in kyphotic angulation progression is a high level of bone oedema coupled with a superior endplate disruption in the affected vertebra [63].

**Fig. 7:**
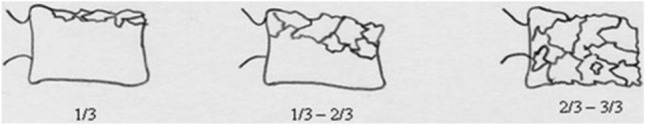
Morphological Modifier II (MM II): Comminution of the vertebral body (Spine Section DGOU [51])

An additional morphological criterion is the dislocation of the fragments. A distinction is made between fragments that are non-dislocated, dislocated < 2 mm, and dislocated by > 2 mm. The part of the vertebral body where the dislocation is located is also differentiated. The dislocation of the fragments in the endplate area is an indication of the expected damage to the adjacent intervertebral disc (Fig. 7).

**Fig. 8:**
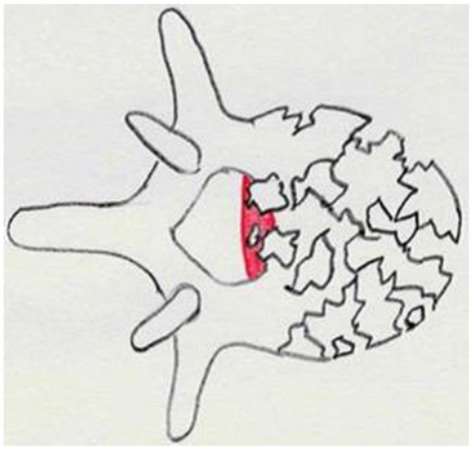
Morphological Modifier III (MM III): Stenosis of the spinal canal (Spine Section DGOU [51])

**MM 3:** Stenosis of the spinal canal: The most constricted area in the axial section of the spinal canal in the affected segment is decisive for the stenosis of the spinal canal due to bony fragments or protrusion of the posterior wall of the vertebral body. The spinal canal’s cross-sectional area is estimated in a horizontal CT section in relation to the upper and lower neighbouring segments in percentages (Fig. 8).

**MM 4:** Extent of disc injury: Traumatic disc injuries do not show a sufficient spontaneous healing tendency [56]. The degree of destruction of the endplate is an indication of the extent of the intervertebral disc injury. If the extent of the disc injury is unclear, an MRI should be considered.

### Indications for non-operative therapy

Non-operative treatment should be performed if there are either general or local contraindications to surgery. These include, above all, serious internal concomitant diseases associated with a greatly increased risk of surgery as well as local reasons such as multisegmental metastases, which make sufficient stabilization impossible, or skin changes that pose a significantly higher risk of infection. In principle, as already mentioned, injuries in which there is no threat of a relevant deformity in the further course can be treated conservatively.

### Based on the AO fracture classification, the following fracture types apply:

#### A0 Minor, non-structural fracture

With this type of fracture, early mobilization with adequate pain therapy and physiotherapy should be take place.

#### A1 Wedge compression (MM 1)

The decisive factor here and with A3 fractures is the extent of kyphosis. With a δEPA < 15–20°, functional therapy can be initiated. In the presence of a δEPA > 15–20°, surgical therapy in the form of instrumentation is advisable to prevent an increase in the kyphosis angle.

#### A2 Split (MM 2, MM 4)

With these fracture types, early mobilization with adequate pain therapy and physiotherapy is possible. An indication for surgery may be a wide separation of the fragments and/or lesion of the adjacent intervertebral disc [64].

#### A3 Incomplete burst (MM 1, MM 2, MM3, MM 4)

With this type of fracture too, the extent of the kyphosis angle is crucial. δEPA < 15–20° and/or scoliosis < 10° can be treated functionally.

#### A4 Complete burst (MM 1, MM 2, MM 3, MM 4)

The same criteria apply here as for A3 fractures.

### Outpatient or inpatient non-surgical treatment

Outpatient treatment of patients with conservative, thoracolumbar spinal injury is possible in cases of functional therapy, mobilized patients and properly adjusted pain therapy. Inpatient admission should happen in case of significant pain symptoms and/or insufficient mobility.

After a short period of rest with adequate pain therapy, rapid mobilization should take place [57–59]. Here close cooperation between doctor, patient, physiotherapist and nurse is crucial. Clinical and radiological controls are required until fracture healing. Where possible, X-ray check-ups after 1, 3, 6 and 12 weeks should be performed while standing, as this is the only way to reliably detect malpositions [60]. It may be useful to supplement these with CT or MRI. If there is an aggravation of the findings in the further course, this can lead to an indication for surgery.

Accompanying measures include sufficient thrombosis prophylaxis, physiotherapy if necessary combined with respiratory gymnastics, and decubitus prophylaxis with adequate pain therapy [61]. Sometimes the use of specific orthoses can be useful [62].

## Chapter 6: Operative treatment

### Introduction

The introduction of rods by Harrington in 1955 formed the base of scoliosis surgery, and it was in the late twentieth century that the first trauma cases were operated more often. Since then, implants and surgical techniques for stabilization of the fractured spine have developed vastly. The goals for surgery however remain the same: to restore the curvature of the spine and to improve quality of life [64].

Strong evidence for surgery still lacks and a clear, robust recommendation regarding treatment (conservative vs operative), and type of surgery (posterior, anterior or combined) cannot be specified [65]. The optimal treatment for patients without neurological deficit and spinal fractures remains debatable, yet patients with spinal fractures type AO B and C and those with neurological deficits do profit from surgery. Further collapsing and kyphosis of the vertebral column may be prevented by dorsal stabilization. If polytraumatized patients can be mobilized earlier because of operative treatment, complications like decubitus or pneumonia could be avoided. In case of traumatic spinal cord injury, advance deterioration can be prevented and even recovery of the neurological status can occur after decompression and operative treatment.

The goal of stabilization can be instrumentation or fusion (spondylodesis). Instrumentation is defined as posterior or anterior stabilization without the definitive fusion of articulation motion segments. Spinal fusion or “spondylodesis” is defined as a permanent fusion of a motion segment. This can be done through either an anterior or a posterior approach. The technique of posterior fusion includes decortication of the interbody joint, placement of autogenous or allogenic bone graft, or use of osteoconductive and/or osteoinductive bone substitutes. Anterior reconstruction is defined by an anatomical restoration of the ventral column with use of implants (cages, ventral instrumentation), grafts or other materials. This can also be performed through a posterior approach [70].

### Operative techniques

When the classification is TLS AO > 5, and in patients with a neurological deficit, surgery is generally advised. Long segmental instrumentation should be used at the upper and middle thoracic spine (above T10). At the thoracolumbar junction and the lumbar spine short segmental stabilization is mostly sufficient, with better clinical outcomes [70–72]. As in most surgical techniques, stabilization of spinal fractures is becoming increasingly possible thanks to minimally invasive techniques.

Dorsal stabilisation by fixation of the noninjured vertebral cranial and caudal of the index fracture is standard. There is growing evidence favouring placement of pedicle screws in the noninjured part of the index fracture as well [66], especially in short-segment stabilized thoracolumbar fractures. In patients older than 70, those with neurological deficits and decompression, or patients with osteoporotic disease, a long trajectory with more screw fixation is advisable. Also, the diameter of the screws is more important than the length of the screw.

Monoaxial implants should be used if no additional anterior stabilization is performed. In contrast, loss of reduction is more likely in patients instrumented with polyaxial screws. Transverse connecting rods can increase stability [70].

Cement augmentation with PMMA (polymethyl methacrylate) cement is a useful tool in patients with reduced bone quality. It is generally not recommended in young patients with a healthy bone stock [70].

The indication to additionally stabilize the fractured vertebral segment anteriorly is still under debate. Excellent results and even long-term data are present on vertebral stenting, balloon-assisted endplate reduction and minimally invasive cementing [67]. Fusing the spine dorsally, combined with dorsal instrumentation, seems unnecessary for fracture treatment.

In cases with unfavourable morphological modifiers (see Chapter 5), anterior stabilization by instrumental implants can be considered. Cages, vertebral stenting and anterior plate fixation are implanted with good, long-term follow-up results. In most cases the additional anterior stabilization can be planned as a second step. Minimally invasive techniques using video-assisted thoracoscopic techniques are provided [68, 69], preventing serious postoperative complications and providing the best stabilization by 360° fixation. This is likewise a successful technique for cases with non-union, for example AO A2 fractures or failing posterior stabilization.

Other techniques like 3D or augmented reality are upcoming and promising developments. Navigation by computer systems, thereby minimizing X-ray radiation and improving pedicle screw placement, is expanding. Robotics and virtual reality will allow spinal surgeons to have real-time, three-dimensional access to images of the spine.

### Complications of operative treatment

The most common complication is malpositioning of screws. The abovementioned techniques with navigation-guided implantation can improve optimal screw placement. Compared to many other trauma operative procedures, the infection rate after open and minimally invasive surgery on the spine is relative low. It is estimated that < 5% of wounds become infected, and thanks to the excellent vascularity of the spine and soft-tissue coverage, treatment with antibiotics is generally effective. Iatrogenic SCI, postoperative bleeding and liquor leakage remain indications for a re-exploration. As minimally invasive techniques are becoming the gold standard, the incidence of infections will probably decrease further.

## Chapter 7: Spinal cord injury

Acute traumatic spinal cord injury (tSCI) is complex and heterogeneous damage, where level of injury, injury severity, duration and degree of spinal cord compression, and blood pressure management seem to influence neurological outcome [74, 84, 91, 94]. Patients with complete thoracic tSCI have a reduced potential for neurological recovery compared with complete cervical tSCI [94], and a trend towards poorer outcome is reported in patients with higher thoracic compared with lower thoracic and thoracolumbar tSCI [93]. A higher-energy injury mechanism, [79] scarcer blood supply of the spinal cord [87], and a narrower spinal canal may play a role in greater tissue disruption in the thoracic region. On the other hand, the potential for neurological recovery in incomplete thoracic, thoracolumbar and cervical tSCI appears to be similar [94]. Nevertheless, neurological outcome after tSCI depends on primary and secondary injury, and mitigating secondary injury represents a key target for intervention in the acute phase [74]. In this regard, early decompressive surgery, arterial blood pressure augmentation and methylprednisolone sodium succinate (MPSS) administration have been suggested as treatment options in the acute phase [74].

### Decompressive surgery and haemodynamics

A recent clinical practice guideline for the management of patients with acute tSCI suggests that early decompressive surgery within 24 h of injury be offered as an option for adult acute tSCI patients regardless of level and severity of injury [80]. Although there is growing evidence supporting early decompression in cervical trauma [78, 81, 84, 85], in the setting of thoracic and thoracolumbar SCI there is still controversy regarding the ideal timing for decompression [95]. A recent meta-analysis did not observe a significant beneficial effect of surgical decompression within 24 h of injury in patients with thoracic and thoracolumbar tSCI [93], whereas a more recent randomized control trial showed that surgical decompression within 24 h of acute traumatic thoracic and thoracolumbar SCI is safe and associated with improved neurological outcomes [82]. A better functional outcome was reported in patients who underwent surgical decompression within 8 h of injury compared to later decompression in both the thoracic and the thoracolumbar spine [96, 97]. The positive effect of early surgery on neurological recovery is more evident in incomplete tSCI [76, 95], as the primary injury in patients with complete injuries may be so severe that no intervention can result in neurological improvement [95]. These observations are in line with previous studies reporting superior neurological recovery in patients with cervical tSCI who underwent surgical decompression within the first 8 h of injury compared to later time windows [78, 84, 85], and in incomplete injuries [84, 85]. Taken together, these data support the “Time is spine” concept, which emphasizes the biological rationale for decompressive surgery as soon as possible after tSCI in order to mitigate secondary injury [73], questioning the suggested time window of 24 h for decompression. Accordingly, it is suggested to perform posterior reduction, decompression and fixation of thoracic and thoracolumbar spinal injuries as damage control surgery aimed to enhance spinal cord perfusion as soon as possible after injury, and which should not be postponed for nonmedical reasons, especially in patients with incomplete injuries. Namely, spinal cord compression from bone fragments, haematoma and dura increases intraspinal pressure (ISP) and results in a drop in spinal cord perfusion pressure (SCPP), which correlates with poor neurological outcome after tSCI [83, 92]. Timing of the decompression remains controversial, but based on recent literature it should take place within 24 h. In case of further neurological deterioration, decompression should be performed immediately.

SCPP not only depends on ISP but also on mean arterial blood pressure (MAP) [92]. Immediate MAP monitoring and management is therefore suggested to prevent hypotension, as tSCI is often additionally complicated by neurogenic shock and/or polytrauma [98]. Recent guidelines suggest a target MAP between 85 and 90 mm Hg for at least 5–7 days after injury [91]. Management of hypoxia, fever and acidosis are suggested to improve local spinal cord metabolism [83], and prophylaxis to prevent deep venous thrombosis should be administered as soon as possible [74]. It is shown that very early surgical decompression is feasible only in patients who are transferred directly from the site of injury to a specialized centre [85], therefore a direct transfer of all tSCI patients from the site of injury to a hospital capable of definitive care is recommended.

### Methylprednisolone sodium succinate

The use of methylprednisolone sodium succinate (MPSS) in tSCI has been a matter of dispute in recent decades because evidence is still lacking. If administration is considered it should be given within the first 8 h, according to the 2017 AOSpine guideline. The dose should be 30 mg/kg IV over one hour followed by an infusion of 5.4 mg/kg per hour for the next 23 h [8]. Contraindications are polytrauma, elderly patients, and patients with complete SCI.

## Chapter 8: Timing of operative treatment

We must differentiate between a spinal fracture with or without neurological deficit.

### Thoracolumbar fractures without spinal cord injury

There are several systematic reviews on timing in thoracolumbar fractures. Bellabarba et al. conclude that, ideally, patients with unstable thoracic fractures should undergo early (within 72 h) stabilization of their injury to reduce morbidity and possibly mortality [99]. Dan Xing et al. conclude the same in a review of 10 studies with 2512 patients. Early stabilization shortened hospital length of stay, intensive care unit length of stay and ventilator days, and reduced morbidity and hospital expenses particularly for patients with thoracic fractures. However, reduced morbidity and hospital expenses were not observed with stabilization of lumbar fractures [100].

In a retrospective study Boakye et al. classified patients as having early (< 72 h) or late (> 72 h) surgery. Early surgery for traumatic thoracic/thoracolumbar fractures was associated with a significantly lower overall complication rate (including cardiac, thromboembolic and respiratory complications) and decreased hospital stay. In-hospital charges were significantly lower ($38,120 difference) in the early surgery group. Multivariate analysis identified time to surgery as the strongest predictor of in-hospital complications, although age, medical comorbidities and injury severity score were also independently associated with increased complications. We reinforce the beneficial impact of early spinal surgery [101].

In another retrospective study Kobbe et al. differentiate between AOSpine A-type and B/C-type injuries in multiple-injured patients. Patients treated within 24 h showed a significantly reduced length of ICU stay by 7 days as compared to those were operated on after 24 h while having a comparable overall injury severity. Furthermore, the length of hospital stay was significantly reduced by 10 days and the prevalence of sepsis was significantly lower. Subgroup analysis showed that the adverse effect of delayed spinal stabilisation is mainly attributable to multiple-injured patients with AOSpine B-/C-type injuries. Regression analysis revealed that in patients with AOSpine A-type spinal injuries, an increased time to spinal surgery was only an independent risk factor for an increased length of hospital stay [108].

Patients with a thoracolumbar fracture without spinal cord injury should be stabilized within 72 h. There is some evidence that multiple-injured patients with AOSpine B/C-type injury should be stabilised even earlier.

### Thoracolumbar fractures with spinal cord injury

As for timing of operative management, high-quality studies comparing early and delayed intervention are lacking. Extrapolating from the evidence in cervical spine injury leads to an assumption that early intervention would also be beneficial for neurological recovery in these patients [102].

For patients in a trauma unit who have a spinal cord injury, the trauma team leader should immediately contact the spinal surgeon on call at the trauma unit or nearest major trauma centre [103]. Patients with neurological deficits caused by traumatic spinal canal stenosis should be treated as an emergency [104].

Fehlings et al. stated that there are currently no standards on role and timing of decompression in acute SCI. They recommend urgent decompression of bilateral locked facets in patients with incomplete tetraplegia or in patients with spinal cord injury with neurological deterioration. Urgent decompression in acute cervical spinal cord injury remains a reasonable practice option and can be performed safely. There is emerging evidence that surgery within 24 h may reduce length of intensive care [105]. Surgical decompression within 24 h of acute spinal cord injury is associated with improved sensorimotor recovery. The first 24–36 h after injury appear to represent a crucial time window to achieve optimal neurological recovery with compressive surgery following acute spinal cord injury [106]. As mentioned in chapter 7, Wilson et al. and Wutte el al. reported a better functional outcome in patients who underwent surgical decompression within 8 h of injury compared to later decompression in both the thoracic and the thoracolumbar spine [95, 96].

Early surgery and severity of initial injury (complete [ASIA A] vs incomplete spinal cord injury [ASIA B-D]) were found to significantly influence the potential for neurological improvement (P 1/4 0.004 and P < 0.0001, respectively) [107].

Thoracolumbar fractures with spinal cord injury should be treated as an emergency. There is some evidence that operating within 24 h results in neurological improvement. In case of further neurological deterioration, decompression should be performed immediately.

## Chapter 9: Osteoporotic fractures

Osteoporotic fractures are reaching epidemic proportions on a global scale. In 2010–2030, the number of elderly with osteoporosis will grow by 32% [113]. The prevalence of vertebral fractures in adults over 40 is at 5.4% and rises to 18% in those over 80. Vertebral compression fracture (VCF) can trigger a vicious cycle of pain and immobility, and can lead to exacerbation of comorbidities, poorer respiratory function and increased risk of death over a five-year period by 72%, or even 90% for very old people over the seven-year monitoring period [114].

For the elderly, a fracture is caused by a small external force: falling from standing height or lifting a moderately heavy load. Nevitt’s bone fragility coefficient can be used to explain the occurrence of a vertebral compression fracture even without an accident [109]. The coefficient is calculated by dividing stress (fall from height, gravity) by bone strength. Fractures from osteoporosis are therefore considered both as an accident and a disease, as dividing the coefficient explains fracture as an accident (fall from standing height), divided by the disease (bone deformation from reduced bone strength) [110].

The vertebral bodies are affected mainly by compressive and to a lesser degree also by tension and stretching forces. Approximately half of the stress comes from the forces of muscles and tendons that hold the body upright, while the other half is caused by the body’s weight. Additional stress is caused by ongoing activities [111].

Osteoporotic vertebral fractures occur in one of three forms: compression fractures, where the height of the entire vertebra is reduced; wedge fractures, where the vertebra collapses in the anterior part (most often in the mid-thoracic spine); and biconcave fractures (fishtail shape), where the vertebra collapses in the middle section (most often in the lumbar part of the spine).

The most frequent are wedge fractures, followed by biconcave, compression, and a combination of all three fracture types. Walking upright compresses vertebrae to the density that according to Nevitt’s coefficient (in the denominator) is demanded by the body mass (in the numerator). This interpretation covers over 30% of vertebral fractures in which the patient does not remember the accident. The latest surveys show that spontaneous fractures in the thoracolumbar spine, which can even be asymptomatic at first, account for over 60% of fractures. MRI uncovers occult fractures, which in traditional radiology would not display any traumatic deformation.

The German Society of Orthopaedics and Trauma Surgery (DGOU) introduced a classification of osteoporotic fractures from OF 1 to 5; see the classification in Chapter 4 [117]:

OF1 represents no fracture but vertebral oedema (MRI).

OF2 fracture, no involvement of posterior wall.

OF3 fracture, distinct involvement of posterior wall.

OF 4 loss of integrity of the vertebral frame or vertebral body collapse.

OF5 fracture with distraction or rotation.

### Treatment

Adequate pain management is mandatory to allow early mobilization, and basic disease treatment is recommended by the WHO guidelines. Long time use of braces in the elderly is not recommended. Regular follow-up should take place.

### Balloon kyphoplasty and vertebroplasty

Balloon kyphoplasty (BK) and vertebroplasty (VP) are minimally invasive augmentation methods that have received critical focus after two articles that cast doubt on their reliability among experts. BK is a minimally invasive surgical procedure to treat pain and correct the kyphotic angle with types A1 and A2 osteoporotic fractures. Using two inflatable balloons, which are introduced transpedicularly, we correct the vertebral deformity and fill the fractures and the cave formed by the inflated balloon in the vertebra using bone cement (i.e. eggshell technique). The literature focuses on pain relief, and patients can raise themselves upright just a few hours after the procedure without any major pain. Indications are:

1. unbearable pain with an acute fracture in the thoracic or lumbar spine
2. tendency for a continuous collapse of a vertebra and additional reduction in height, visible on X-rays in an upright position
3. persistent acute pain for more than three weeks after the fracture

With osteoporotic fractures it is sometimes difficult to differentiate an acute fracture from past chronic changes and acute pain from other comorbidities, therefore MRI examination of the spine is recommended before BK. VP is another augmentative method, where cement is injected transpedicularly into the fractured corpus of the vertebra; however, without inflatable balloons the Cobb angle of kyphosis has less correction than with BK. Some authors see VP as a developmental early stage of BK.

Both methods have described complications, such as bone cement extravasation through the hairline fractures from the vertebral body into neighbouring anatomic structures (spinal canal, vena cava, aorta), compression of neurological structures and venous embolisms. By using the eggshell technique with BK, complications can often be avoided. Comparing results one year after VP and BK, it has been established that the fracture of an adjacent vertebra occurs with advancing osteoporosis and depends on the biomechanics of kyphosis. After either method it is a rare complication, with no statistically significant differences in incidence. These results suggest that the adjacent vertebra would fracture eventually, even without the augmentation procedure. VP an BK offer a comparable rate of pain relief [112].

In 2009 two sham control studies were published, sowing doubt on the effectiveness of these augmentation methods. Consequently, the number of procedures conducted declined for a few years, resulting in reduced patient survival rates. For five years, elevated mortality risk in VCF patients was observed [115]. Later studies have proven the effectiveness of both augmentation methods, and numerous recommendations once again place them among effective methods for treating acute fracture pain and chronic pain, and provenly improving quality of life for seniors who suffer from fractures. Unlike non-surgical therapy, BK is more effective at pain relief, back-related disability, and quality-of-life improvement [116].

Hybrid constructs are recommended for osteoporotic spinal fractures with a burst component (according to OF classification OF3 and especially OF4 fractures) with minimally invasive posterior transpedicular instrumentation and some augmentation techniques as described above [117].

## Chapter 10: Implant removal

There is no definitive position regarding the removal of implants after operative fixation of a TL fracture; neither is there a clear answer as to whether implants should be removed routinely, how long after the initial fracture stabilization, nor as to whether an implant should only be removed when it starts causing problems for the patient. The reason for this lies in the risks that any operative procedure presents. One year after implantation, degenerative changes in the disc generally cause a reduction of the intervertebral disc space, arthrosis of the zygapophysial joints and reduced movement of the fixated segment, and it is reported in the literature that after 8 years, adjacent fractures occur in a third of cases where the implant was not routinely removed [118]. Greater benefit lies in removing the implant in younger patients and with longer fixations that take into account several moving segments at least 12 months after the first procedure, and after the fracture has radiologically healed [122]. In cases of ankylosing spondylitis and with older patients, it is not recommended to remove the implant without a justified clinical reason [123].

Even though most patients with symptomatic and asymptomatic implants feel a subjective improvement after removal, and even though operative removal of the implant is related to a low percentage of operative complications, the literature recommends individualized treatment of each patient, especially an in-depth discussion. Even if we discover a single broken screw, it is not necessary to remove it. Objective reasons for implant removal are infection, migration or endangered neurological structures, and issues related to the spine’s flexibility with long fixations. In the end, however, the patient’s decision must be respected [122].

## Conclusion/summary

1. If a spinal injury is suspected, the patient must be immobilized and transferred directly from the site of injury to a specialized centre.
2. Adequate imaging is essential for the further treatment of the patient.
3. Classification based on the CT-scan is mandatory for further decision-making.
4. Conservative treatment is possible if no further deterioration is expected. Early mobilization combined with adequate pain management is essential. Follow-up must take place.
5. Operative treatment can prevent further deterioration and allows early mobilization in unstable fractures. Various techniques are available.
6. Early decompression and maintenance of an adequate mean arterial pressure are essential for recovery from spinal cord injuries.
7. Osteoporotic fractures are both an injury and a disease. Adequate therapy for the basic disease is mandatory.
8. Implant removal is an individual patient-based decision.
